# Effects of kinesiotaping on disability and pain in patients with rotator cuff tendinopathy: double-blind randomized clinical trial

**DOI:** 10.1186/s12891-022-05046-w

**Published:** 2022-01-26

**Authors:** Fatima Zahrae Taik, Samia Karkouri, Latifa Tahiri, Ilham Aachari, Jihad Moulay Berkchi, Ihsane Hmamouchi, Redouane Abouqal, Hanan Rkain, Fadoua Allali

**Affiliations:** 1 Department of Rheumatology, Tangier-Tetouan-El Hoceima University Hospital, Tangier, Morocco; 2 Faculty of Medicine and Pharmacy, Abdelmalek Essaadi University, Tangier, Morocco; 3 Department of physical medicine and rehabilitation, El Ayachi Hospital, Ibn Sina University Hospital, Rabat, Morocco; 4 Faculty of Medicine and Pharmacy, Mohammed V University, Rabat, Morocco; 5 Department of Rheumatology B, El Ayachi Hospital, Ibn Sina University Hospital, Rabat, Morocco; 6 Laboratory of Biostatistics, Clinical Research and Epidemiology (LBRCE), Faculty of Medicine and Pharmacy, Mohammed V University Rabat, Rabat, Morocco; 7 Laboratory of Physiology, Faculty of Medicine and Pharmacy, Mohammed V University, Rabat, Morocco

**Keywords:** Kinesiotaping, Shoulder, Rotator cuff tendinopathy, Pain, Disability

## Abstract

**Background:**

Kinesiotaping (KT) is widely used in several musculoskeletal disorders particularly in shoulder pain. However, literature shows controversial results regarding the effect of KT on shoulder pathology. The aim of this study was to assess the clinical effects of KT in the short term on rotator cuff tendinopathy (RCT).

**Methods:**

A randomized controlled double-blind clinical trial was conducted. The sample consisted of 50 subjects (25 per group). Patients were randomly assigned to the KT group (to receive therapeutic KT application) or to the placebo group (to receive sham KT application). Taping was applied every 4 days, a total of three times during the study period. We assessed the patients at baseline, at the end of taping period (D12), and at one-month post-taping (D30). Primary outcome was assessed through the Arabic version of the Disabilities of the Arm, Shoulder and Hand questionnaire (DASH). Secondary outcomes were assessed through Visual Analogue Scale (VAS) for pain intensity at rest (VASr), during active movement (VASm), and at night (VASn).

**Results:**

There were no significant differences between the two groups in the demographic and clinical characteristics and the pre-test scores. Results of repeated measures ANOVA showed significant improvement in DASH scores and in VAS for pain (at rest, during active movement and at night) from D12 in both groups. The use of ANCOVA, controlling for pre-test scores, showed no significant differences between groups, except for VASm at D30.

**Conclusion:**

This study showed that the standardized therapeutic KT used for shoulder pain was not superior to a sham KT application in improving pain and disabilities in patients with RCT.

**Trial registration:**

The study was retrospectively registered on Pan African Clinical Trial Registry (identification number: PACTR202007672254335) on 21/07/2020. https://pactr.samrc.ac.za/TrialDisplay.aspx?TrialID=12200

## Background

Rotator cuff tendinopathy (RCT) is a frequent reason for consultation; it represents between 44 and 65% of chiropractic visits for shoulder pain [1]. RCT also impacts on patients’ functionality, sleep, quality of life and work performance at a considerable socio-economic cost [2–5]. RCT is the result of degenerative lesions that are the consequence of intrinsic and extrinsic factors, including anatomical and biomechanical dysfunctions in addition to age-related injuries (tendon degeneration, poor vascularization) and overuse in relation to specific sports and professions [6–8]. This is a spectrum of pathologies that include simple tendonitis, calcifications and tendon tears. Medical treatment, described as first-line treatment, includes pharmacological and non-pharmacological measures. Kinesio Taping (KT) is one of the conservative treatments proposed for rotator cuff disease as well as other musculoskeletal disorders [9–11]. KT is a practice inspired by traditional Japanese medicine, developed by Kenzo Kase in 1979 [12]. It is a flexible taping method done with a special material that is impermeable and non-degradable in water, without any added chemicals substances [12]. Adhesive bandages produce directed traction on skin, which may have a positive effect on muscle and joint systems by reducing pressure on subcutaneous mechanoreceptors. Although the pathophysiological mechanism is not fully understood, it is thought that KT also improves blood and lymphatic circulation and reduces pain and muscle tension [13]. However, there is conflicting research in the literature regarding the effect of KT on RCT [14–19]. The aim of our study was to evaluate the effect of KT on RCT in the short term.

## Methods

### Study design

This was a double-blind randomized controlled clinical trial, conducted in collaboration between the Rheumatology B and Physical Medicine and Rehabilitation Departments of the El Ayachi hospital of Salé, between May 2019 and February 2020, which aimed to evaluate the effects of KT on RCT in the short term as compared to a sham KT application. The study was approved by the Biomedical Research Ethics Committee of Rabat. The study was retrospectively registered on Pan African Clinical Trial Registry on 21/07/2020, identification number: PACTR2020076722543.

### Population

We included in our clinical trial patients who fulfilled the following inclusion criteria: 1) age between 20 and 60 years, 2) shoulder pain before 150° of active elevation in any plane, 3) pain during resisted external rotation, abduction or empty can test and 4) positive signs of conflict (Neer’s or Hawkins sign) on clinical examination. Patients were excluded if they had: 1) progressive dermatological pathology contraindicating the application of an adhesive shoulder bandage, 2) history of surgery, fracture or dislocation of shoulder, 3) local corticosteroid infiltration in the previous 6 months, 4) reproduction of symptoms during the cervical screening examination, 5) cervical radiculopathy, or 6) hyperpilosis that may impede the application of KT. Patients who met the clinical inclusion criteria and had none of the exclusion criteria were subsequently subjected to an ultrasound examination of their shoulders by a senior rheumatologic sonographer (LT). Ultrasound examination was performed to characterize anatomical lesions (simple tendonitis, bursitis, calcification, or partial tear) and to exclude patients with a transfixing tear on the affected side. Based on the hypothesis of a 12% improvement in our primary outcome measure in the KT group as compared to the placebo group, with a 5% alpha risk and a power of 95%, the total sample size consisted of 50 participants. 12% improvement was identified as success criteria because the Clinically Important Difference (CID) of the Disabilities of the Arm, Shoulder and Hand questionnaire (DASH) (which ranges from 0 to 100) is 10–12 point [20].

All participants provided written informed consent to participate in the trial.

**Fig. 1:**
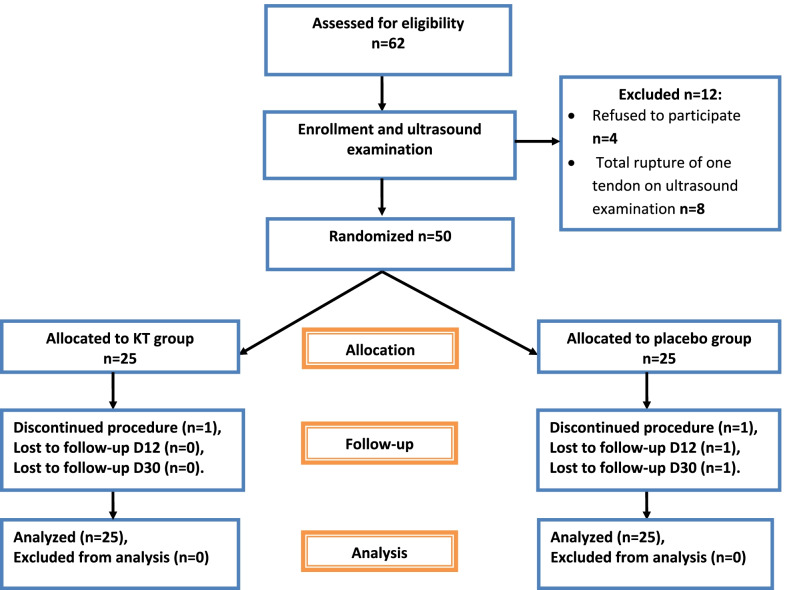
Flow diagram for the randomized study

Details of subjects included and excluded from the study (from inclusion to analysis) are shown in Fig. 1.

### Procedure and taping technique

Subjects were randomized (using a random-number generator) into two groups to receive either 1) standardized therapeutic KT for shoulder pain (KT group, *n* = 25) or 2) sham KT application (placebo group, *n* = 25). Tapes were applied by a certified KT practitioner (SK) while the first author (FZT), who was blinded to the patients’ groups (patients were not undressed during evaluation), evaluated the outcomes. Patients were instructed to maintain normal daily activities but to avoid unusual physical effort or sports activity. Analgesic therapy was not permitted except for severe pain and had to be recorded if taken.

Patients in the KT group benefited from the application of two pink strips of KT under 25% tension (25% tension was obtained by removing 20% of the length of the strip which corresponds to the length of the muscle on which the strip will be placed, measured directly on the patient, and by stretching it at the time of application) exerted at the center of the adhesive tape (the anchoring points were not taut): 1) a Y-shaped strip (obtained by cutting the strip longitudinally in the middle from one side to produce two tails) at the deltoid, from its insertion to its origin. The first tail of the band was applied to the anterior region of deltoid, maintaining the patient’s arm in 60–80° horizontal abduction and complete external rotation, while the second tail was applied to the posterior region of deltoid, with the arm in 20–30° horizontal adduction and complete internal rotation; and 2) an I strip at supraspinatus muscle, applied from its insertion to its origin, on a position of lateral cervical flexion to the opposite side with the arm held behind the back.

**Fig. 2:**
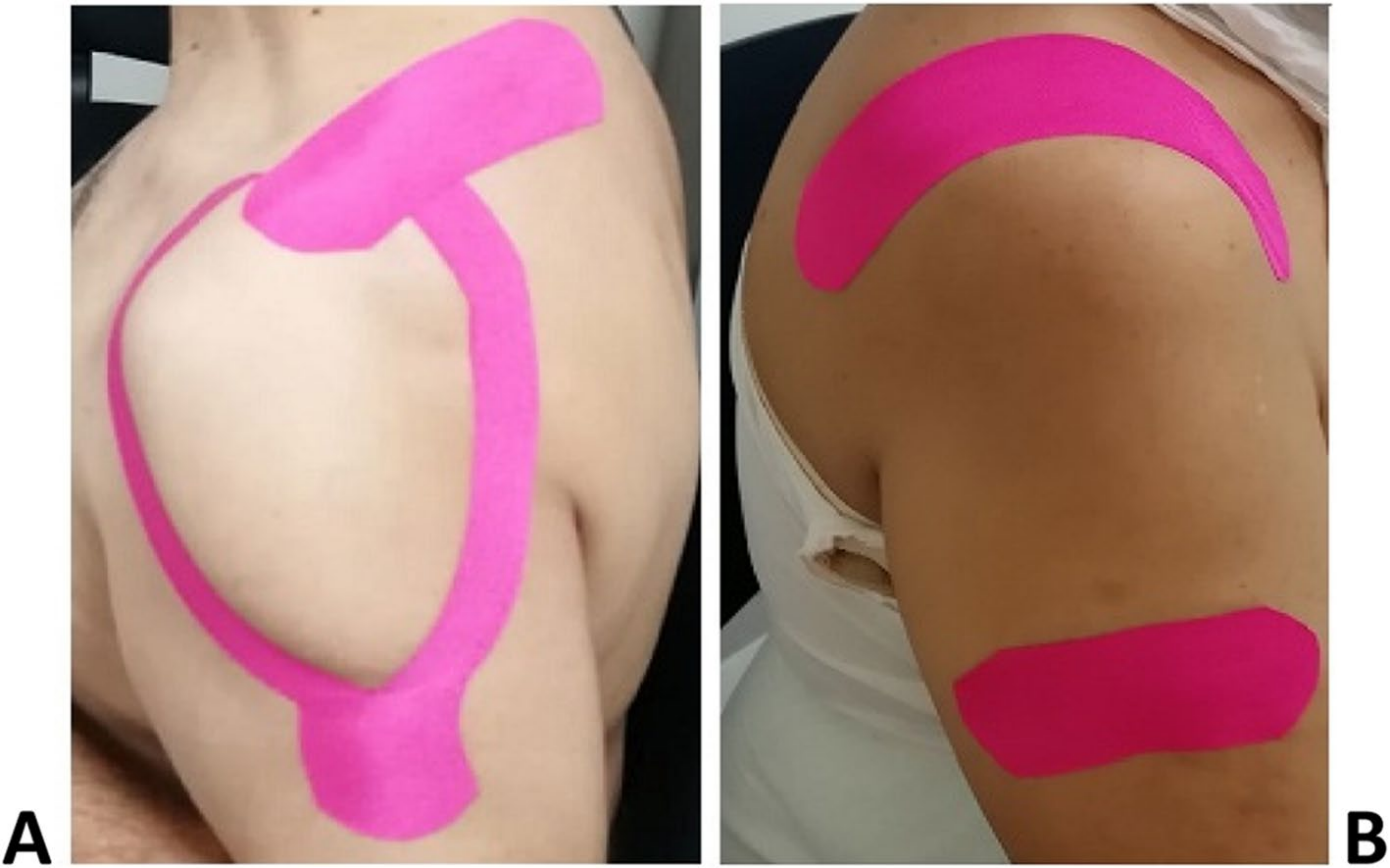
**A** KT application in KT group. **B** Sham KT application in placebo group

Two tension-free pink I-strips were applied to patients in the placebo group: 1) one 12-cm-long strip at the acromioclavicular joint in the sagittal plane 2) and one 10-cm-long strip at the distal insertion of the deltoid in the horizontal plane, with the patient’s arm held in neutral position. For the placebo group, the taping technique used has no rationale; we simply applied KT strips to the sites most often indicated by the patients as the location of pain. Figure 2 illustrates the taping technique for both groups.

The patients in both groups had a total of three applications, 4 days apart, initiated just after the baseline assessment. Patients were asked to keep the strips in place as long as possible between applications, except in the event of itching, and, even if a strip came off spontaneously, they should attend their next appointments normally.

The subjects were informed that two different taping techniques were applied, but they were not given any further details about the taping procedure. We consider that blindness of the study was respected since all the patients received the same nature of tape (KT), the same number of strips (2 strips), the same number of applications and the same color of strip. Concerning the tension of strips, we do not consider that this compromised blindness since even strips applied without additional tension provides a sensation of skin tension. This was confirmed by all subjects who reported that they did not know their group assignment at the end of the study.

### Outcome measures

Our study participants were evaluated at baseline (D1), at the end of taping (D12) and 1 month after the first application (D30). Our primary outcome measure was the function of the upper limb as assessed by the Arabic version of the Disability of the Arm, Shoulder and Hand (DASH) questionnaire [21]. The Arabic version of the DASH questionnaire is a validated score to assess upper limb disabilities in various diseases. The questionnaire contains 30 questions: 21 items refer to difficulties encountered in performing functional activities using the arm, shoulder and/or hand; five questions assess the degree of painful symptoms (activity-related pain, tingling, stiffness and weakness); and four questions assess the impact on quality of life (social activities, work, sleep) and the psychological impact. The scale ranges from 0 (no disability) to 100 (greatest disability) [21]. Secondary outcome measures were pain (using a 10 cm visual analog scale) at rest (VASr), pain during active movement (VASm) and pain at night (VASn).

### Statistical analysis

A descriptive analysis of the entire population was conducted using headcount and percentage for qualitative variables, and median and standard deviation for quantitative variables if they were normally distributed; otherwise they were expressed in medians and quartiles (the distribution of the variables was evaluated by the Kolmogrov-Smirnov test). Pre-treatment comparison between groups was performed with Student’s T-test for independent groups (for quantitative variables with normal distribution), the Mann-Whitney test (for quantitative variables with asymmetric distribution), and the Chi-2 test (for qualitative variables). Repeated measures of ANOVA were used to determine any significant changes in the tested variables in each group after treatment. The analysis of covariance (ANCOVA) was used to determine if there were significant differences in the post-treatment scores between the two groups, with pre-treatment scores used as co-variables in the analysis. Data were analyzed using SPSS_21.0. Test results were estimated as significant if *p* < 0.05. The analysis was performed with an intention-to-treat approach.

## Results

Both study groups were comparable in terms of demographic and clinical characteristics and inclusion scores (Table 1).

**Table 1:** Patients’ demographic and clinical characteristics

	KT group *n* = 25	placebo group *n* = 25	*p*-Value
Age^a^	57.2 ± 6.62	57.12 ± 8.88	0.97
Female gender^b^	23 (92%)	23 (92%)	> 0.99
Affected side			0.15
• Right^b^	10 (40%)	15 (60%)	
• Left ^b^	15 (60%)	10 (40%)	
Symptoms duration (month)^3^	6 [2.5–12]	12 [4.5–12]	0.33
Echography findings			
• Tendinitis^b^	6 (24%)	5 (20%)	0.86
• Bursitis^b^	9 (36%)	9 (36%)	> 0.99
• Calcification^b^	3 (12%)	5 (20%)	0.44
• Partial tear^b^	7 (28%)	8 (32%)	0.75
Baseline DASH^a^	41.6 ± 10.84	44.5 ± 12.67	0.39

Values are given as mean ± standard deviation (^a^), frequency (^b^), or median and quartiles (^c^)

*DASH* Disabilities of the Arm, Shoulder and Hand

We started the analysis with repeat-measures ANOVA that showed statistically significant improvement in the DASH score after intervention for both the KT group (*p* = 0.001) and the placebo group (*p* < 0.001). Similar results were observed for VASr, VASm and VASn (Table 2).

**Table 2:** Baseline and post-taping scores for tested variables in KT and placebo groups

	KT group *n* = 25				Placebo group *n* = 25				ANCOVA
	baseline	Day 12	Day 30	***p***	baseline	Day 12	Day 30	***P***	***p-***Value
DASH	41.6 ± 10.84	33.16 ± 12.87	32.65 ± 13.9	0.001	44.55 ± 12.67	34.62 ± 16.68	34.65 ± 13.9	< 0.001	0.8
VASr	1.48 ± 1.75	0.72 ± 1.3	0.6 ± 1.08	0.012	1.64 ± 1.65	1.2 ± 1.35	1.12 ± 1.5	0.019	0.45
VASm	4.6 ± 0.81	3.16 ± 1.37	2.6 ± 1.41	< 0.001	4.6 ± 0,91	3.28 ± 1.64	3.44 ± 1.78	< 0.001	**0.049**
VASn	3.6 ± 1.97	2.68 ± 1.93	2.16 ± 1.79	< 0.001	3.8 ± 1.61	2.8 ± 1.63	2.48 ± 1.87	< 0.001	0.78

*DASH* Disabilities of the Arm, Shoulder and Hand, *VASr* Visual Analog Scale for pain at rest, *VASm* Visual Analog Scale for pain during active movement, *VASn* Visual Analog Scale for pain at night

Post-hoc analysis showed that a significant decrease in the DASH score and pain was observed from D12 onwards. The ANCOVA analysis, using pre-treatment scores as co-variables, did not show a statistically significant difference between the two groups in terms of improvement in the DASH score (*p* = 0.8), VASr (*p* = 0.45), VASn (*p* = 0.78). However, there was a statistically significant difference in the decrease of the VASm (*p* = 0.049) between the two groups at D30 (Table 2).

## Discussion

In this study, we explored the short-term effects of KT on RCT in comparison to a placebo taping. The results of our study indicate that therapeutic and sham KT produced similar effects on disability and pain. Indeed, after taping, the DASH score improved (at both assessment times) by 21–22% for the KT group and 23% for the placebo group with no statistically significant difference between the two groups. Similarly, there was an improvement in VASr (50–60% for the KT group and 27–32% for the placebo group) and VASn (26–40% for the KT group and 27–35% for the placebo group) with no statistically significant difference between the two groups. However, there was a statistically significant difference in decrease of VASm between the KT group (44%) and the placebo group (26%) at 1 month (*p* = 0.049).

Our results are partially in agreement with other studies. Thelen et al. had already shown in 2008 [22] that, with the use of taping, pain and disability measures were similar between KT and placebo groups, and they had concluded that the use of KT for improving pain or disability in young patients with suspected RCT is not supported [22]. Kocyigit et al. demonstrated that KT and sham taping generated similar effects in terms of pain and Constant Scores in shoulder subacromial impingement syndrome [23]. However, Shakeri et al. reported a significantly greater decrease in the DASH score after 1 week of KT in a treatment group than in a control group, and concluded that KT can be used to decrease disability of arm, shoulder and hand related to shoulder impingement syndrome [24]. A similar finding was reported by Simsek et al. who showed that pain with movement and the DASH scores were significantly improved in the therapeutic group at the fifth day (*p* < 0.01) in comparison to the placebo group, and they suggested that KT application in association to exercise therapy is more efficient than exercise therapy alone for the treatment of shoulder impingement syndrome [14].

Shoulder range-of-motion (ROM) was not assessed in our study. KT seems to have no effect on the ROM in several studies [14, 23, 24] while Thelen et al. showed immediate improvement in pain-free shoulder abduction in a KT group [22]. In addition to clinical findings, some studies had assessed the sonographic and electromyographic effects of KT. Kaya et al. performed diagnostic ultrasound assessment for supraspinatus tendon thickness and indicated that ultrasound findings were similar in a KT group compared to a manual therapy group [25]. Lin et al. reported, after electromyographic assessment of subjects without shoulder injuries, that KT generated changes in proprioception and scapular muscle activity [26].

Our study produced an interesting finding: sham KT application also improved pain and disabilities in patients with RCT. This can be explained by the fact that, in addition to the placebo effect, the KT band, whatever the application technique, can create a sensory stimulation. Semsek el al. had suggested that sham KT was able to stimulate the subcutaneous mechanoreceptors and, thus, activate the motor neurons, which can explain the obtained clinical result [14]. However, the sham taping (surgical hypoallergenic flexible tape applied in the same way as KT) used by Kocyigit et al. also produced a significant decrease in VAS for nocturnal pain and Constant Score similar to the KT group [23]. They proposed that applying the sham taping in the same manner as the KT group may activate the gate control [23]. Therefore, we consider that our control group (sham KT application) was not appropriate for a placebo group. We recognize that it was a limitation of our study.

Further studies should include a non-taping group to ensure that the obtained results were due to the effect of the KT and not to the natural course of the disease.

Other limitations should be noted: 1) the majority of the patients (58%) had reported a decrease in their daily physical activity, even though they were instructed to maintain a normal daily routine; we consider this as a possible source of confusion. 2) Our sample was heterogeneous; we included patients with bursitis, simple tendonitis, calcification and partial tear. Subgroup analysis was not possible due to the small sample size. In this sense, further studies will be necessary to identify the profile of patients most likely to respond to KT.

## Conclusion

This study showed that the standardized therapeutic KT used for shoulder pain was not superior to a sham KT application in improving pain and disabilities in patients with RCT. Further studies are required to define the potential benefit of KT and the patients most likely to respond to it.

## Data Availability

The datasets used and/or analyzed during the current study are available from the corresponding author on reasonable request.

## Abbreviations

ANOVA: Analysis of variance
ANCOVA: Analysis of covariance
CID: Clinically Important Difference
DASH: Disabilities of the Arm, Shoulder and Hand questionnaire
KT: Kinesiotaping
RCT: Rotator cuff tendinopathy
ROM: Range-of-motion
VAS: Visual Analogue Scale
VASr: Visual Analogue Scale for pain intensity at rest
VASm: Visual Analogue Scale for pain during active movement
VASn: Visual Analogue Scale for pain and at night
